# Automatic activation of motor programs by object affordances in patients with Parkinson's disease

**DOI:** 10.1016/j.neulet.2009.07.033

**Published:** 2009-09-29

**Authors:** Hiroaki Oguro, Robert Ward, Martyn Bracewel, John Hindle, Robert Rafal

**Affiliations:** a Wolfson Centre for Clinical and Cognitive Neuroscience, School of Psychology, Bangor University, Bangor, Wales, United Kingdom; b Department of Neurology, Shimane University, Faculty of Medicine, Japan; c North West Wales NHS Trust, United Kingdom; d School of Medical Sciences, Bangor University, United Kingdom

**Keywords:** Parksinon's disease, Affordance, Kinesia paradoxical, Basal ganglia, Motor control

## Abstract

Clinical observations of *kinesia paradoxica* and freezing in patients with Parkinson's disease suggest that the automatic activation of motor programmes by visual stimuli may not require intact basal ganglia function, and that an increased sensitivity to such object affordances may contribute to some symptoms of the disease. Employing a paradigm that measures the degree of interference from object affordances on voluntary actions, we confirm that activation of object affordances are preserved in Parkinson's disease, but find no evidence that there is an increased sensitivity to the effects of object affordances on voluntary action.

Gibson [2] first suggested that some object features automatically activate an ‘affordance’ for action. For example, the appearance of a handle affords its grasping and automatically activates a motor program to grasp. Observations that salient visual stimuli can facilitate movement [1,4] in patients with Parkinson's disease (PD), and even trigger rapid movements in otherwise frozen individuals – *kinesia paradoxica* – suggests that automatic action activation by object affordances may not require intact basal ganglia function. The fact that visual stimuli can also interfere with voluntary action and induce freezing [3] also raises a question as to whether automatic motor program activation by object affordances might actually be disinhibited in PD.

We tested this hypothesis, employing a paradigm [5] that measures the degree of interference from object affordances on voluntary actions. In this paradigm (Fig. 1) an automatic affordance is activated by the presentation of a picture of a frying pan, which participants are instructed to ignore, with the handle oriented to afford grasping with either the left or right hand. Then an arrowhead pointing left or right is presented in the middle of the frying pan, and participants press a key with the right hand if the arrow points rightward or with the left hand if the arrow points leftward. In healthy individuals, viewing of the frying pan automatically primes a motor response by the hand for which it affords a grasping response. This results in faster reaction times to arrows requiring the same response as the affordance primed by the picture, and slower reaction times to arrows requiring a different response than the affordance primed by the picture. This difference in reaction, or compatibility effect, provides a measure of the strength of motor priming by object affordances.

Seventeen patients with idiopathic PD, 11 men and 6 women ranging in age from 58 to 77 (mean 65.5, SD = 4.9), participated after giving informed consent under a protocol approved by NHS and University research ethics committees, and conforming to the Declaration of Helsinki. All participants had capacity to give informed consent. All patients had symptoms of hypokinetic Parkinson's disease, with disease duration ranging from 10 to 151 months (mean = 67.2 months, SD = 49.7) (see Table 1). Motor symptoms at the time of testing were assessed with 27 motor subset items (18–31) from the Unified Parkinson's Disease Rating Scale (UPDRS). UPDRS scores ranged from 5 to 32 (mean 18.1, SD = 8.9). Testing was done while patients were taking their usual medication. None had dyskinesias at the time of testing. Thirteen sex and age-matched control participants were recruited through the School of Psychology community panel. Both groups were screened to excluded dementia, psychiatric disease, stroke, head injury and other neurological abnormalities. Patients were assessed by the Mini-mental State Examination (MMSE): all > 26.

Stimuli appeared on a white background of a video monitor placed 57 cm in front of seated participants, who made responses on the keyboard of a Macintosh Powerbook G3 computer, using Psyscope version 1.2.5 software to present stimuli, and to measure of reaction times and error rates. After participants initiated each trial by pressing the space bar, an image of a “prime” object appeared: a colored picture of a large fry pan with the handle angled toward the observer and oriented, with equal probability, toward either the left or right hand of the participant. (Fig. 1) These images subtended 21.7° (horizontal) and 9.2° (vertical) of viewing angle and were presented centrally, in color upon a white background. The orientation and depth of the handle simulated an apparent affordance for grasping with either left or right hand. After 1200 ms a small arrowhead pointing equiprobably to left or right was projected on the centre of the pan instructing the participant to press any key on the left side of the keyboard with the left hand or any key on the right side of the keyboard with the right hand. The target appeared in the centre of the display, superimposed over the prime, subtending a viewing angle of 2.9° (horizontal) and 1.1° (vertical). The relation between the target and the orientation of the fry pan handle was random such that the affordance activated by the prime was equally likely to be compatible or incompatible with the required response.

After a practice block of 30 trials, the experiment comprised one block of 200 trials in a within-subject, two-factor design. The factors consisted of response (left or right hand) and prime-target compatibility (the left/right orientation of the handle in the prime in the same (compatible response) or opposite (incompatible response) direction to the target response).

Participants were instructed to maintain gaze on the central fixation marker, to ignore the fry pan and to respond to the arrow by making a left or right response as quickly as possible. Feedback tones were given on incorrect trials.

After excluding errors (2% for patients and 1% for controls), RT distributions were iteratively trimmed to within 3SDs of the condition mean for each participant. Mean reaction time was calculated for each participant for compatible and incompatible prime-target conditions and submitted to an ANOVA. There was a trend for patients to have longer reaction times (mean = 598 ms, SE = 29 ms) than controls (mean = 508 ms, SE = 3 ms), *F*[1,28] = 3.2, *p* = 0.085). There was a main effect of prime-target compatibility (*F*[1,28] = 6.261, *p* = 0.018), with no interaction between group and compatibility *F*[1,28] < 1). The compatibility effect was 18 ms for the control group and 17 ms for the Parkinson's disease patients. The size of the compatibility effect induced by the affordance did not correlate with UPDRS motor disability scores (Spearman's rho = −.14). For four of the patients who had reported episodes of freezing, the affordance compatibility effects were distributed throughout the range of the Parkinson's patient group Table 2.

We found that object affordances primed motor responses in patients with Parkinson's disease as effectively as in healthy controls. There was no evidence, in the current group of PD patients, that object affordance effects were disinhibited. While the affordances that we measured experimentally were for manual and not for locomotor activity, we did not find that affordance compatibility effects were related to freezing or to the degree of motor disability. We conclude that visual cues may help Parkinsonian patients to bypass neural processes needed for endogenous motor programming and facilitate their actions in the visual environment. However, perceptual capture is not, in itself, a factor contributing to motor disability.

## Figures and Tables

**Fig. 1:**
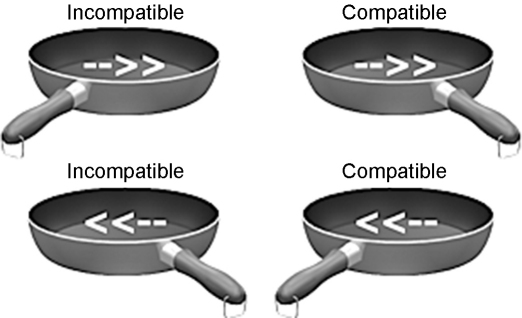
The four conditions shown occurred with equal probability. The fry pan was displayed for 1200 ms before the target arrow, instructing either a left or right hand response, was projected on the center of the pan.

**Table 1:** Patient details.

Patient	Age/sex	Disease duration (mo. since diagnosis)	UPDRS score
1	68/M	36	17
2	69/F	15	21
3	68/F	25	23
4	69/F	62	13
5	58/F	151	16
6	77/M	123	31
7	61/M	64	20
8	58/M	18	5
9	66/M	66	32
10	68/F	10	30
11	61/M	42	16
12	63/M	32	10
13	62/M	135	32
14	63/M	42	10
15	64/M	144	7.5
16	71/F	42	9.5
17	67/M	135	32

**Table 2:** Mean RT in ms (SE in parentheses) in compatible and incompatible prime conditions for Parkinson's disease patients and controls.

	Parkinson patients	Controls
Compatible condition	589 (44)	499 (18)
Incompatible condition	606 (38)	517 (20)
Compatibility effect^a^	17 (12)	18 (5)

a Compatibility effect = RT incompatible − RT compatible.
